# Synovial Regulatory T Cells Occupy a Discrete TCR Niche in Human Arthritis and Require Local Signals To Stabilize FOXP3 Protein Expression

**DOI:** 10.4049/jimmunol.1500391

**Published:** 2015-11-11

**Authors:** David Bending, Eirini Giannakopoulou, Hannah Lom, Lucy R. Wedderburn

**Affiliations:** Infection, Inflammation and Rheumatology Section, Institute of Child Health, University College London, London WC1N 1EH, United Kingdom

## Abstract

Although there is great interest in harnessing the immunosuppressive potential of FOXP3^+^ regulatory T cells (Tregs) for treating autoimmunity, a sizeable knowledge gap exists regarding Treg fate in human disease. In juvenile idiopathic arthritis (JIA) patients, we have previously reported that atypical CD25^+^FOXP3^−^ Treg-like cells uniquely populate the inflamed site. Intriguingly, their proportions relative to CD25^+^FOXP3^+^ Tregs associate with arthritis course, suggesting a role in disease. The ontogeny of these FOXP3^−^ Treg-like cells is, however, unknown. In this study, we interrogated clonal relationships between CD4^+^ T cell subsets in JIA, using high-throughput TCR repertoire analysis. We reveal that FOXP3^+^ Tregs possess highly exclusive TCRβ usage from conventional T cells, in blood, and also at the inflamed site, where they are clonally expanded. Intriguingly, the repertoires of FOXP3^+^ Tregs in synovial fluid are highly overlapping with CD25^+^FOXP3^−^ Treg-like cells, indicating fluctuations in FOXP3 expression in the inflamed joint. Furthermore, cultured synovial Tregs rapidly downregulated FOXP3 protein (but not mRNA), and this process was prevented by addition of synovial fluid from JIA patients, through an IL-6–independent mechanism. Our findings suggest that most Tregs arise from a separate lineage from conventional T cells, and that this repertoire divergence is largely maintained under chronic inflammatory conditions. We propose that subsequent Treg expansions at the inflamed site creates an environment that leads to competition for limited resources within the synovium, resulting in the destabilization of FOXP3 expression in some Tregs.

## Introduction

Forkhead box protein 3^+^ regulatory T cells (Tregs) are thought to be critical for the maintenance of tolerance (1–3) and the control of excessive inflammation, yet Tregs are frequently increased in numbers in autoimmune arthritis of childhood, juvenile idiopathic arthritis (JIA) (4, 5). JIA has served as a unique and useful human model to understand Treg dynamics and interactions for several reasons: 1) access to the anatomical site of disease gives precious insight into the mechanisms involved in the perpetuation of chronic inflammation in humans; and 2) patients with JIA can have diverging clinical outcomes, allowing correlation between immunological findings and disease prognosis. Two striking findings from recent research have demonstrated that both an increased frequency of FOXP3^+^ Tregs (4, 5) and coexpression of CD25 and FOXP3 (6) are associated with the milder disease courses in JIA. These data strongly suggest that treatment strategies to boost/augment Treg numbers could be fruitful for treating JIA, or indeed other autoimmune conditions. However, studies of Tregs often consider the phenotype and function of the Treg population as one homogenous group, negating the fact that Tregs likely express a diverse repertoire of TCRs, signals through which are essential to their function (7). TCR diversity is driven in part because of genetic recombination of Variable, Diversity and Junctional gene segments (8) within the *TCRB* gene family locus. This recombination generates a unique DNA sequence that can be used as a barcode to track the fates of T cell clones during immune responses.

The huge diversity of the TCR repertoire has previously been a barrier to meaningful analysis in human disease; however, recent technological advances mean high-throughput repertoire analysis is feasible and cost-effective. Recent exhaustive repertoire sequencing of human peripheral blood suggests a highly diverse repertoire with a lower bound limit of at least a million unique clonotypes (9). However, the repertoires involved in human arthritis are predicted to be more clonal, based on heteroduplex TCR analysis in isolated CD4/CD8 T cells (10). TCR repertoires are likely to be important in autoimmunity because it has been shown in JIA that T cell clonotype expansions mirror the HLA class I (CD8 expanded) or class II (CD4 expanded) association of arthritis (10). In addition, recent insight in animal models of arthritis suggests that certain TCRs can transfer arthritis even in the presence of Tregs (11). Furthermore, repertoire analysis has started to yield unprecedented insight into the heterogeneity of human memory Th cells that respond to common pathogens or vaccines (12), giving us a greatly enhanced picture of developmental relationships between CD4^+^ T cell subsets.

Several observations in JIA have questioned both the developmental relationships between Tregs and conventional T cells (Tcons) and the relationships between different Treg subsets at the inflamed site. We have previously reported that Tregs and pathogenic Tcons show an inverse reciprocal relationship at the inflamed site in JIA (5). This could imply a developmental relationship between these two subsets, which could be easily determined only through analysis of TCR repertoires. Analysis in mice has suggested that the repertoires of Tregs and Tcons are diverse, with only a partial overlap in the periphery (13–15). The relationship in humans remains to be determined. Second, we also reported that Tregs can adopt atypical phenotypes at the inflamed site: in addition to “classic” CD25^+^FOXP3^+^ Tregs, CD25^−^FOXP3^+^ and CD25^+^FOXP3^−^ Treg-like populations exist, both of which display demethylation at the Treg-specific demethylated region (TSDR) (6). The origin of these populations is unclear, but we have reported that Treg coexpression of CD25 and FOXP3 is important for disease progression (6), which means understanding Treg fate and provenance at inflamed sites is important for understanding disease.

In this study, we aimed to determine the clonal relationships between Tcon and Treg subsets both in blood and in the inflamed site of JIA patients, using the immunoSEQ TCRB survey platform (16, 17). Our results demonstrate a near-complete separation of Tregs from Tcon TCR repertoires in both blood and the inflamed site in JIA. Synovial T cell repertoires contain numerous expanded clones; however, synovial Tregs cluster more closely with blood Tregs than Tcons from the inflamed site, implying that a significant proportion of Tregs in the joint arise from Tregs in the blood. Intriguingly, the repertoires of FOXP3^+^ Tregs in synovial fluid (SF) are highly overlapping with atypical CD25^+^FOXP3^−^ Treg-like cells, implying dynamic FOXP3 expression in vivo. This is illustrated by the finding that FOXP3^hi^ Tregs rapidly downregulate FOXP3 protein in the absence of SF, highlighting that local signals are essential for sustaining FOXP3 expression. Our findings give unprecedented insight into the makeup of the TCR repertoires involved in a human autoimmune response, as well as the dynamic relationships between Tcon and Treg subsets in vivo.

## Materials and Methods

### Human samples

Twenty-five patients with JIA contributed SF mononuclear cells (SFMCs; see Supplemental Table I) or PBMCs, and 60 patients contributed SF to this study. All fulfilled the International League of Associations for Rheumatology classification criteria (18). Nine healthy control volunteers donated PBMCs, all with no known autoimmune or genetic conditions. Written informed consent was received from all human participants before inclusion in the study, with informed parental consent where appropriate. The study had full ethical approval from the local research ethic committee (95RU04). SFMCs and PBMCs were prepared by density gradient centrifugation using Lymphoprep (Axis-Shield) with SF samples undergoing pretreatment with hyaluronidase (10 U/ml; Sigma). Samples were cryopreserved until use. SF pools were made from 10 to 15 JIA individuals and treated for 30 min with 10 U/ml hyaluronidase before aliquoting and freezing at −80°C. SF was added to cultures at the specified percentage of the final well volume.

### Abs and reagents

The following directly conjugated Abs were used in flow cytometry: BV711 or FITC-conjugated CD4 [OKT4 (Biolegend) or RPA-T4 (eBioscience)], BV711- (clone A019D5; Biolegend) or FITC-conjugated CD127 (eBioRDR5; eBioscience), PE- (BD Biosciences) or BV421-conjugated (Biolegend) CD25 (MA251), allophycocyanin-conjugated FOXP3 (236A/E7; eBioscience) or PE-Cy7–conjugated FOXP3 (PCH101; eBioscience), V450-conjugated active CASPASE3 (C92-605; BD Biosciences), PE-conjugated p-(Y705)STAT3 (4/P-STAT3; BD Biosciences), PE-conjugated p-(Y694)STAT5 (47/STAT5; BD Biosciences), PE-conjugated CTLA-4 (14D3; eBioscience). Recombinant human IL (rhIL)-2 (Roche), IL-6, rhIL-7, rhIL-15 (Peprotech), and rhIL-6 (BD Biosciences) were used at the concentrations indicated in the figure legends. Tocilizumab (TOC; anti–IL-6R) and infliximab (anti–TNF-α) were used at a final concentration of 10 μg/ml. Cycloheximide (Sigma) was used at a concentration of 50 μg/ml.

### Flow cytometry

Flow cytometry was performed with directly conjugated Abs as previously described (6). A fixable Blue LIVE/DEAD dye (Life Technologies) was used to exclude dead cells from analysis. FOXP3 and CTLA-4 (total) staining was performed using the eBioscience FOXP3 staining buffers. For staining of phosphorylated proteins, BD Fix Buffer I and BD Phosflow perm buffer III were used according to manufacturer’s instructions. In brief, sorted Tregs (at 5 × 10^5^/ml in 100 μl RPMI 1640) were incubated for 15 min with either SF or 10 ng/ml rhIL-6 before addition of 100 μl BD Fix Buffer I and incubated for 10 min at 37°C. Cells were washed extensively in PBS, then permeabilized by addition of 1 ml ice-cold Perm Buffer III and incubation for 30 min on ice. Cells were washed three times and then stained for PE–anti–p-STAT3 or PE–anti–p-STAT5. Data were collected on the LSR II, FACS Aria, or FACSCalibur flow cytometers (all BD Biosciences). Flow cytometry data were analyzed using FloJo (Tree Star) software.

### Cell sorting

For live cell sorting, cells were pre-enriched for CD4^+^ T cells using a negative selection enrichment kit (STEMCELL Technologies). Cells were stained for CD4, CD25, and CD127, before filtering and adding DAPI or propidium iodide (PI). Cells were sorted on a FACS Aria (BD Biosciences) cell sorter as follows: Treg: CD4^+^CD127^lo^CD25^−^DAPI/PI^−^; Tcon: CD4^+^CD127^hi^CD25^lo^DAPI/PI^−^. Sorting of fixed and FOXP3-stained cells was performed as previously described (6), with the following modification: cells were collected into a PBS solution containing 10 mM HEPES (Sigma).

### DNA, RNA extraction, and quantitative PCR

DNA was extracted using a modified phenol protocol as previously described (6). RNA was extracted using the PicoPure kit (Life Technologies). Typically, 2.5 × 10^4^ sorted cells or cultured cells were washed once with PBS and then once with PBS containing 50 mM EDTA (Sigma) before lysing in the RNA extraction buffer and incubating at 42°C for 30 min, before centrifugation of the lysate and freezing. RNA was extracted according to the manufacturer’s instructions and converted to cDNA using random hexamer primers as previously described (6). cDNA was amplified using SYBR Green Mastermix (Bio-Rad) with primers to GAPDH (Qiagen), *ACTB* (For: 5′-AGA TGA CCC AGA TCA TGT TTG AG-3′; Rev: 5′-AGG TCC AGA CGC AGG ATG-3′), or *FOXP3* (primer sequences For: 5′-ACC TGG AAG AAC GCC ATC-3′; Rev: 5′-TGT TCG TCC ATC CTC CTT TC-3′) on the Rotor-gene 6000 Corbert thermocycler (Qiagen) using the following cycling conditions: 95°C 5 min, then 40 cycles of 95°C 30 s, 60°C 30 s, 72°C 30 s. SYBR green emission data and melt curves were collected. *FOXP3* gene expression is displayed relative to *GAPDH* or *ACTB* using the 2^−ΔCt^ method.

### TCRB sequencing and analysis

A total of 100–400 ng genomic DNA from sorted T cell subsets was processed by Adaptive Biotechnologies (Seattle, WA) on the immunoSEQ human TCRB survey platform (16, 17). In brief, input DNA was amplified in a two-step multiplex PCR in which the first PCR amplified the CDR3 region of T cell genomes and the second PCR added adaptor sequences compatible with Illumina Next Generation Sequencing (NGS) platform. Sequencing was performed using Illumina’s Next Generation Sequencing platform. Using a baseline developed from a suite of synthetic templates, we used primer concentrations and computational corrections to correct for the primer bias common to multiplex PCRs. Raw sequence data were filtered based on the TCRβ V, D, and J gene definitions provided by the IMGT (ImMunoGeneTics) database (http://www.imgt.org) and binned using a modified nearest-neighbor algorithm to merge closely related sequences and remove both PCR and sequencing errors. Data were analyzed using the Adaptive Biotechnologies immunoSEQ Analyzer. Analysis was based on unique, productive reads at the nucleotide level. The degree of repertoire clonality was defined as the reciprocal of normalized entropy [entropy was calculated by the Shannon entropy index (19)] and then normalized by dividing the entropy score by the log (2) of the unique productive reads, thereby allowing comparison of samples that vary by the total number of reads detected. The repertoire overlap between two samples (A and B) was calculated using the immunoSEQ analyzer. The overlap statistic is simply the percent of shared sequences for all pairwise comparisons. That is, the sum of shared sequences for sample A and sample B, divided by the sum of sequences for sample A and sample B.

### Cell culture

Cells were cultured on 96-well V-bottom plates (CoStar) in 10% FBS RPMI 1640 at 37°C 5% CO_2_. Cells were cultured at a final concentration of 5 × 10^5^/ml for the time points indicated. For SF and JIA peripheral blood (PB) Tregs, 2.5 × 10^4^ cells were cultured in 50 μl. For control Treg, 2.5 × 10^4^ (Fig. 3, Supplemental Fig. 2) or 5 × 10^4^ (Fig. 6) cells were used. In some experiments, cultures were supplemented with 10 or 50% SF, recombinant cytokines (IL-2, IL-7, IL-15), or cytokine blocking Abs (anti-IL-6R or anti-TNFα) as stated in the figure legends.

### Luminex

Sixty SFs from JIA patients were analyzed for IL-6 and IL-10 expression levels using cytokine multiplex assay as previously described (20).

### Statistics

Statistical analysis was performed on Prism 5 for Mac (GraphPad) software. For clarity, statistical tests and error bar descriptions are included in the figure legends. A *p* value <0.05 was deemed statistically significant.

## Results

### The TCR repertoires of Tregs and Tcons show high exclusivity in blood and SF of JIA patients

**FIGURE 1.**
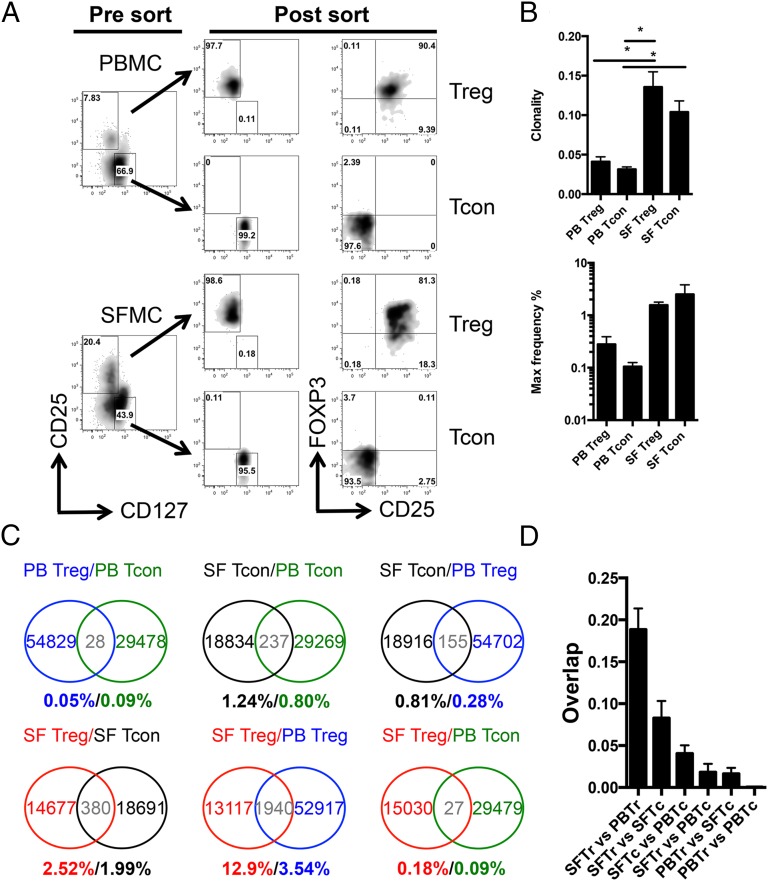
The TCR repertoires of Tregs and Tcons show high exclusivity in blood and SF of JIA patients. PBMCs and SFMCs taken at the same time from JIA patients were enriched for CD4^+^ T cells and then stained for CD4, CD127, and CD25. (**A**) *Left panels* show CD25 versus CD127 staining before sorting. Tregs (CD4^+^CD127^lo^CD25^hi^) and Tcons (CD4^+^CD127^hi^CD25^lo^) were sorted from both anatomical compartments; sort purities based on CD127 and CD25 expression are shown in the *middle panels*. A small sample of sorted cells was stained for FOXP3 (*right panels*). Genomic DNA was extracted from purified T cells and analyzed using the immunoSEQ TCRB survey platform (Adaptive Biotechnologies). (**B**) Graphs depict the clonality (*top panel*) and frequency of the most abundant clone (*bottom panel*) in the TCR repertoires of the four sorted subsets (*n* = 4 individual JIA patients). Bars represent mean ± SEM. Statistical testing by one-way ANOVA. *p < 0.05. (**C**) Venn diagrams depict the sharing of unique clones (at nucleotide level) between each sorted T cell subset from one of the four JIA patients (see Supplemental Fig. 1 for patients 2–4). The percentage figure represents the percentage of sequences shared as a total of that T cell compartments repertoire. Blue represents PB Treg, green represents PB Tcons, black represents SF Tcons, and red represents SF Tregs. Shared unique sequences are depicted in gray. These data are representative of four individuals. (**D**) Bar graph displaying overlap (as calculated in *Materials and Methods*) among the four T cell populations. *n* = 4. Data are representative of four JIA patients, the cells were sorted, and DNA was extracted in three independent experiments but analyzed in two separate runs on the immunoSEQ platform. Tc, Tcon; Tr, Treg.

We decided to use the immunoSEQ platform (Adaptive Biotechnologies) (16, 17) to perform high-throughput TCR repertoire analysis of CD4^+^ T cell subsets in JIA patients (Fig. 1), because this platform can use genomic DNA as a template and has been successfully used by other groups to address TCR repertoires in health and disease (12, 21). A total of 100,000–200,000 Tregs and Tcons were isolated from the blood and SF of the same patients, based on cell-surface expression of CD25 and CD127 (Tregs: CD4^+^CD127^lo^CD25^hi^, Tcons: CD4^+^CD127^hi^CD25^lo^; Fig. 1A), to high purity. Genomic DNA was extracted and run on the immunoSEQ TCRB survey platform. TCR repertoires at the inflamed site were significantly more clonal compared with the peripheral blood, as expected (Fig. 1B). In keeping with this, the inflamed site contained numerous expanded clones, with the most abundant clones being ∼1–2% in the SF, compared with 0.1% in the blood compartment (Fig. 1B). We next compared the sharing of nucleotide sequences, which would indicate clonal sharing among different repertoires (Fig. 1C, Supplemental Fig. 1). Almost no sharing of clones was detected between blood Tregs and blood Tcons or SF Tcons. Similarly, blood and SF Tcon repertoires also shared very few sequences. Interestingly, the repertoires of the SF and PB Tregs showed the greatest frequency of clone sharing (mean of 12.7% of SF Treg clones shared with PB Tregs, *n* = 4). When the whole repertoire size was taken into account (not just unique sequences but their relative abundance between two populations), the overlap of PB and SF Tregs was found to be almost 0.2, and only a small overlap of SF Tregs and SF Tcons was found, suggesting that SF Treg cluster most strongly by cell lineage (Treg) and only weakly by anatomical site (SF) (Fig. 1D).

### Tregs occupy a discrete TCR niche from effector T cells at the inflamed site regardless of FOXP3 expression patterns

**FIGURE 2.**
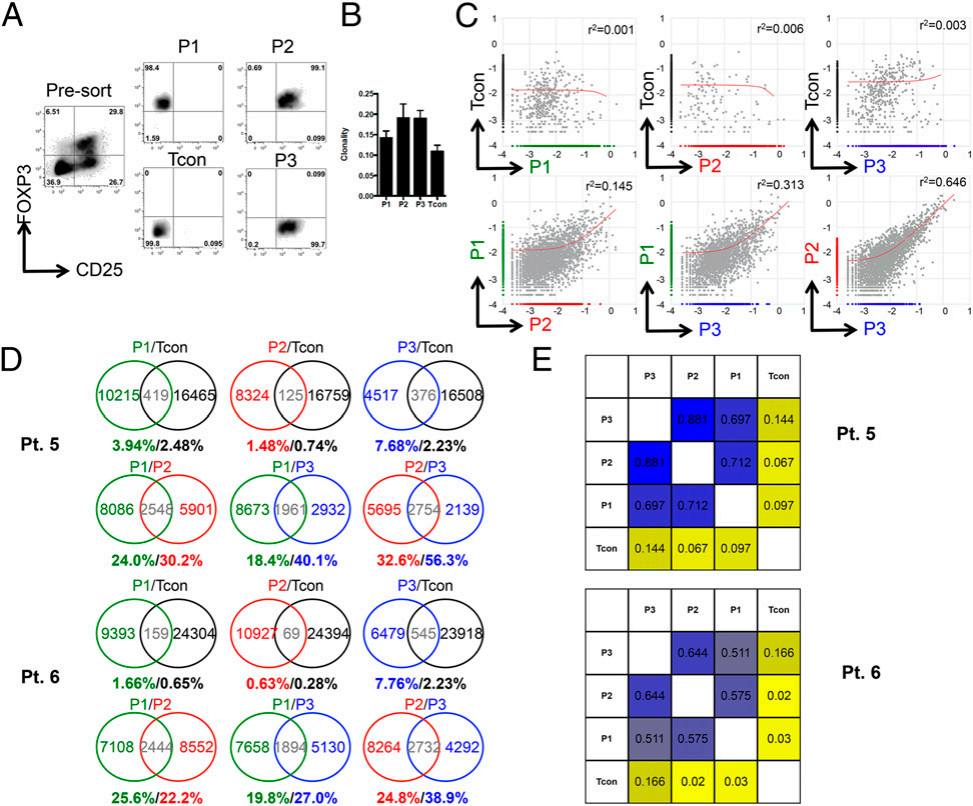
Tregs occupy a discrete TCR niche from Tcons at the inflamed site regardless of FOXP3 expression patterns. SFMCs from a JIA patient were stained for CD4, CD127, CD25, and FOXP3. (**A**) *Left graph* depicts CD25 versus FOXP3 staining (gated on CD4^+^CD127^lo^ T cells) in the presort population. Three Treg and one Tcon population were then sorted as shown: P1: CD4^+^CD127^lo^CD25^lo^FOXP3^hi^; P2: CD4^+^CD127^lo^CD25^hi^FOXP3^hi^; P3: CD4^+^CD127^lo^CD25^hi^FOXP3^lo^; and Tcon: CD4^+^CD127^hi^CD25^lo^FOXP3^lo^. Purities of the sorted population from patient 5 are shown. (**B**) Clonality scores were calculated using the immunoSEQ analyzer software and are shown for the four populations (*n* = 2). Bars represent mean ± SEM. (**C**) Graphs depict the frequency of unique and shared clones between the different Treg and Tcon subsets. Unique clones are placed on the *x*- or *y*-axis and are coded as follows: green, P1 Tregs; red, P2 Tregs; blue, P3 Tregs, black, Tcons; and shared clones are in gray. Correlation was calculated using Pearson’s correlation. Axis numbers represent log (10) scale. (**D**) Venn diagrams depict the sharing of unique clones (at nucleotide level) among the four isolated T cell subsets in two separate patients. The figure represents the percentage of unique clones shared between the two repertoires as a total of the unique clones found in that T cell compartments repertoire. Green represents P1 Treg, red represents P2 Tregs, blue represents P3 Tregs, black represents Tcons, and shared unique sequences are depicted in gray. (**E**) Heat map showing degree of repertoire overlap in two JIA patients. These data are from two independent experiments.

We have recently reported that the Treg signature in SF is atypical, and that CD127 and CD25 expression are not sufficient to identify the entire Treg footprint in SF due to the fact that CD25 and FOXP3 expression are frequently dissociated (6). We therefore explored the relationships and origins of these atypical Treg populations at the inflamed site. To do this, SFMCs were stained for CD4, CD25, and CD127, then fixed and stained for FOXP3, before sorting into their respective populations: P1: CD25^−^FOXP3^+^, P2: CD25^+^FOXP3^+^, P3: CD25^+^FOXP3^−^, and Tcon: CD127^hi^CD25^−^FOXP3^−^ (Fig. 2A). Genomic DNA was extracted and run on the immunoSEQ human TCRB platform. Summary of the sequence reads obtained from genomic DNA extracted from fixed human T cells is shown in Table I. Clonality was slightly increased in Treg populations compared with Tcons but was comparable with the findings achieved with unfixed cells in Fig. 1B (Fig. 2B). The overlap at the nucleotide level was then interrogated between the repertoires of the four populations (Fig. 2C, 2E). The repertoires were first visualized in graphical form based on the frequency of a given clone in each repertoire. This permitted correlation by Pearson’s coefficient for the sharing of clones between two repertoires (Fig. 2C). As can be seen in patient 5, clone sharing and correlations were very weak between all three Treg repertoires and Tcons, but the repertoires of each Treg associated strongly with each other, with the strongest correlation between P2 and P3 (*r*^2^ = 0.646). These data are summarized in Venn diagram form for two JIA patients (Fig. 2D), revealing that typically between 20 and 50% of unique TCR clones were shared between Treg populations, but only 2 and 7% with the SF Tcon, of which P3 Tregs showed the most overlap. When total repertoire overlap was analyzed, taking into account the frequency of the TCRB reads shared, the strong clustering of the Treg repertories was strikingly apparent, with, on average, >0.5 repertoire overlap between the Treg populations (Fig. 2E). In fitting with the results in Fig. 1, repertoire overlap between P1 (mean = 0.064) and P2 (mean = 0.044) Tregs was minimal with Tcons, again highlighting the near exclusivity of the Treg and Tcon repertoires at the inflamed site. P3 Tregs showed some overlap with Tcons (0.155), which could possibly reflect Tcon contaminants within this population or possibly represent Tregs that have converted to Tcons in vivo. The divergence of Tcon and Treg repertoires is strikingly illustrated when the 30 most abundant clones within patient 1 are analyzed (see Supplemental Table II). As can be seen, 16 of 20 of the most abundant 20 clones were exclusive to the P1, P2, and P3 repertoires. Together, these results strongly suggest that Treg clones are expanded at the inflamed site, but that their expression patterns of CD25 and FOXP3 are in flux.

**Table I.** Sequencing depth summary for patient 5 (Fig. 2)

Sample Name	Total	Unique	Productive Total	Productive Unique	Out of Frame Total	Out of Frame Unique	Has Stop Total	Has Stop Unique
P1 Treg	874505	13530	690085	10634	176697	2680	7723	216
P2 Treg	891752	10822	723079	8449	162974	2197	5699	176
P3 Treg	778669	6287	630511	4893	143825	1309	4333	85
Tcon	552247	21268	442549	16884	100383	4021	9315	363

### A subpopulation of synovial Tregs exhibits unstable FOXP3 protein expression in vitro

**FIGURE 3.**
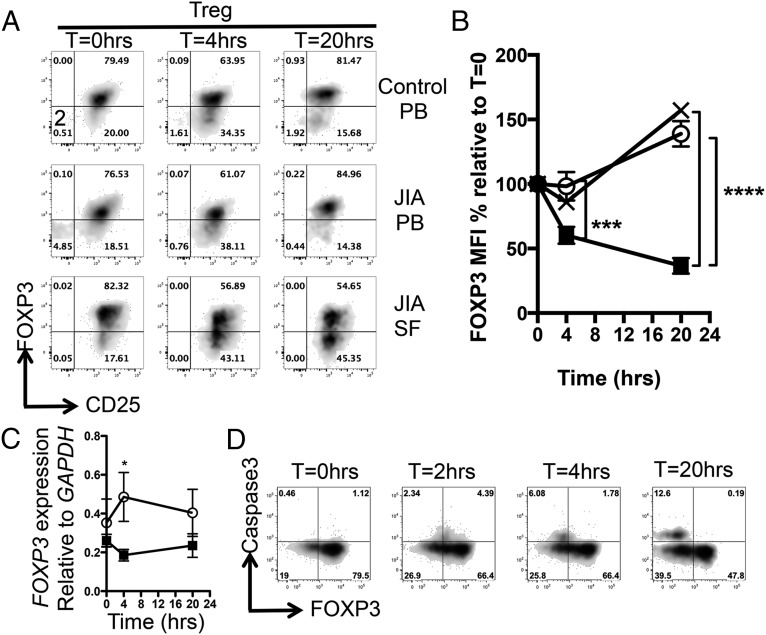
A subpopulation of synovial Tregs exhibits unstable FOXP3 protein expression in vitro. Tregs (CD4^+^CD127^lo^CD25^hi^) and Tcons (CD4^+^CD127^hi^CD25^lo^) were isolated from the blood of healthy control subjects and JIA patients, or SF of JIA patients. Tregs were cultured in media for the indicated time points. (**A**) At times T = 0, T = 4, and T = 20 (for Tregs), cells were stained for CD25 and FOXP3 and analyzed by flow cytometry (A and **B**), or RNA extracted and analyzed for expression of *FOXP3* (**C**). (B) Graph summarizes FOXP3 protein levels calculated using the median fluorescence intensity (MFI) at the indicated time points and normalized to time 0; control Tregs: *n* = 5 (○), JIA PB Tregs: *n* = 3 (×), JIA SF Tregs: *n* = 8 (▪). ***p < 0.001, ****p < 0.0001. (C) The *FOXP3* expression levels relative to *GAPDH* in control (○, *n* = 3) or SF Tregs (▪, *n* = 5) over the 20-h culture period. Bars represent mean ± SEM. Statistical analysis was performed by two-way ANOVA. *p < 0.05. (A and B) Data are representative of at least three independent experiments. (**D**) SF Tregs were stained for active Caspase 3 and FOXP3 at the indicated culture time points and analyzed by flow cytometry. Data are representative of at least three individuals in two independent experiments.

We hypothesized that the expansion of Treg clones within the confined joint environment would likely lead to intraclonal competition for resources such as MHC and/or FOXP3/CD25 maintenance signals, for example, IL-2, with the result being that some clones may downregulate expression of FOXP3 and/or CD25 in vivo. We hypothesized that instability in FOXP3 expression would be revealed by in vitro culture of SF Tregs. To investigate this, we isolated Tregs from SF, or the blood of healthy control subjects and JIA patients, based on CD127 and CD25 expression (Fig. 3A) and cultured them overnight; the expression of FOXP3 and CD25 was analyzed at T = 0, 4, and 20 h. Within the SF Tregs, a FOXP3^lo^ population was observed at 4 h, which was FOXP3^−^ by 20 h. This was not as apparent in healthy control Tregs or JIA PB Tregs. This was a highly reproducible finding, with a significant downregulation in FOXP3 protein at both 4- and 20-h time points (Fig. 3B). Interestingly, although *FOXP3* mRNA levels were lower at 4 h in SF Tregs compared with controls, this was not true at T = 0 or T = 20 h, suggesting that FOXP3 downregulation was largely due to protein loss, rather than transcriptional downregulation. Furthermore, addition of cycloheximide resulted in downregulation of FOXP3 protein in control Tregs, but not SF Tregs, suggesting that FOXP3 protein is rapidly lost in some SF Tregs (Supplemental Fig. 2). Importantly, this protein loss was not due to blockade of the Ab recognition site, because similar results were obtained with either the 236A/E7 or PCH101 anti-FOXP3 clones, which are raised against different epitopes (data not shown). FOXP3 downregulation was not explained by apoptosis, because the majority of the FOXP3^lo^ compartment was negative for active caspase 3 at various time points during the culture (Fig. 3D).

### Addition of SF, but not common γ-chain cytokines, stabilizes Treg FOXP3 protein expression

**FIGURE 4.**
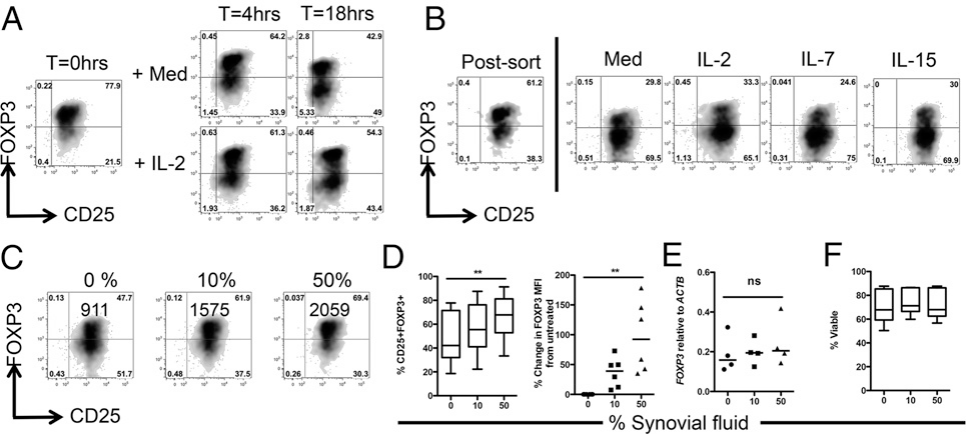
Addition of SF, but not common γ-chain cytokines, stabilizes Treg FOXP3 protein expression. (**A**) SF Tregs (CD4^+^CD127^lo^CD25^hi^) were sorted and then cultured for 0, 4, or 18 h in the presence or absence of 100 U/ml rhIL-2 before analysis of CD25 and FOXP3 expression by flow cytometry. Data are representative of at least three experiments. (**B**) SF Tregs (CD4^+^CD127^lo^CD25^hi^) were sorted and then cultured for 0 or 18 h in the presence or absence of 100 U/ml rhIL-2, 20 ng/ml IL-7, or 20 ng/ml IL-15 before analysis of CD25 and FOXP3 expression by flow cytometry. Data are representative of two independent experiments. (**C**) SF Tregs were isolated and cultured for 4 h in the presence of medium alone, or medium supplemented with 10 or 50% final concentration of SF obtained from JIA patients. Displayed are flow cytometry plots showing CD25 versus FOXP3 expression in the various cultures. (**D**) Summary graph of (C) showing the percentage CD25^+^FOXP3^+^ live cells (*left*) or percentage change in FOXP3 median fluorescence intensity from untreated Tregs (*right*) in six individual JIA patients. Data are representative of two independent experiments. (**E**) In parallel to protein analysis in (C), *FOXP3* expression relative to *ACTB* was assessed by RT-PCR, *n* = 4. (**F**) The frequency of viable cells in the cultures from (C) was determined based on uptake of a fixable Live/Dead dye. Bars represent the medians. Statistical analysis by Friedman test with Dunn multiple comparison (**p ≤ 0.01). ns, not significant.

IL-2 is a known transcriptional activator of FOXP3 expression in Tregs. However, we previously reported that IL-2 alone was insufficient to restore FOXP3 expression in CD25^+^FOXP3^lo^ Tregs isolated from SF (6). Similarly, we found that IL-2, at both early and late time points (Fig. 4A), or IL-7 and IL-15 (Fig. 4B) could not prevent formation of a FOXP3^lo^ Treg population. Similarly, the immunosuppressive cytokines IL-10 and TGF-β could not rescue the phenotype (data not shown).

Given that the majority of Tregs in SF express FOXP3, we hypothesized that a balance of factors at the inflamed site may be important for optimal FOXP3 expression. To investigate the effect of the local environment on SF Treg FOXP3 downregulation, we made a pool of SF from JIA patients and then added them to cultures at 0, 10, or 50% final concentration. As shown in Fig. 4, addition of SF to the cultures had a significant effect on the frequency of CD25^+^FOXP3^+^ Tregs [Fig. 4C, 4D; median frequencies 42.1% (0% SF), 55.5% (10% SF), and 67.8% (50% SF), respectively] and positively changed the levels of FOXP3 protein [Fig. 4D, *right panel*; median increase 39.1% (10% SF) and 92.4% (50% SF) respectively]. *FOXP3* mRNA was analyzed in parallel; interestingly, no differences in transcriptional activity were detected, once again inferring that loss of FOXP3 was principally driven by degradation of the transcription factor (Fig. 4E). Importantly, the effect of SF on FOXP3 expression was not related to improved cellular viability, because addition of SF did not significantly alter the frequency of live cells in the cultures [Fig. 4F; median viable cells 67.8% (0% SF), 71.2% (10% SF), and 68.0% (50% SF), respectively].

### SF is a potent STAT3 activator in Tregs

**FIGURE 5.**
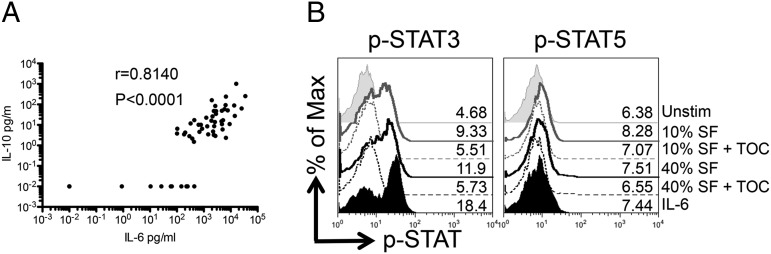
SF is a potent STAT3 activator in Tregs. Sixty SFs from JIA patients were analyzed for IL-10 and IL-6 levels using the Luminex platform. (**A**) IL-6 and IL-10 levels show a strong positive correlation (*r* = 0.8140, Spearman’s rank, *n* = 60, *p* < 0.0001). (**B**) Tregs were isolated from healthy control subjects and stimulated either with medium alone (gray filled histograms) or 10% SF pool (gray line), 10% SF in the presence of 10 μg/ml TOC (gray dashed), 40% SF (black line), 40% SF in the presence of 10 μg/ml TOC (black dashed) or 10 ng/ml IL-6 (black filled histograms) for 15 min before fixation and staining for p-STAT3 or p-STAT5. Numbers indicate the median fluorescence intensity of p-STAT. Data are representative of two independent experiments.

Given that SF appeared to promote FOXP3 expression, we next investigated key signaling pathways engaged by soluble factors within SF. SF is known to be enriched for both proinflammatory (IL-6, IL-12, TNF-α, and IL-1β, which are the current targets of biologics) and anti-inflammatory factors like IL-10 (20). Using the Luminex platform, we determined that IL-6 and IL-10 were the most consistently detected cytokines found within SF, and interestingly, these were strongly positively correlated (Fig. 5A), implying a degree of homeostatic regulation within the joint.

Both IL-6 and IL-10 are activators of the STAT3 pathway, which is thought to negatively regulate Treg differentiation (22, 23). We confirmed that our pool of SF was able to strongly activate STAT3 in Tregs (>200% increase; Fig. 5B), but only very weakly STAT5 (∼15–20% increase). Neutralization of IL-6 signaling using the therapeutic mAb to the IL-6R TOC abrogated the ability of SF to activate STAT3, indicating that IL-6 was the predominant STAT3-activating cytokine.

### IL-6R signaling by SF can positively regulate Treg phenotype

**FIGURE 6.**
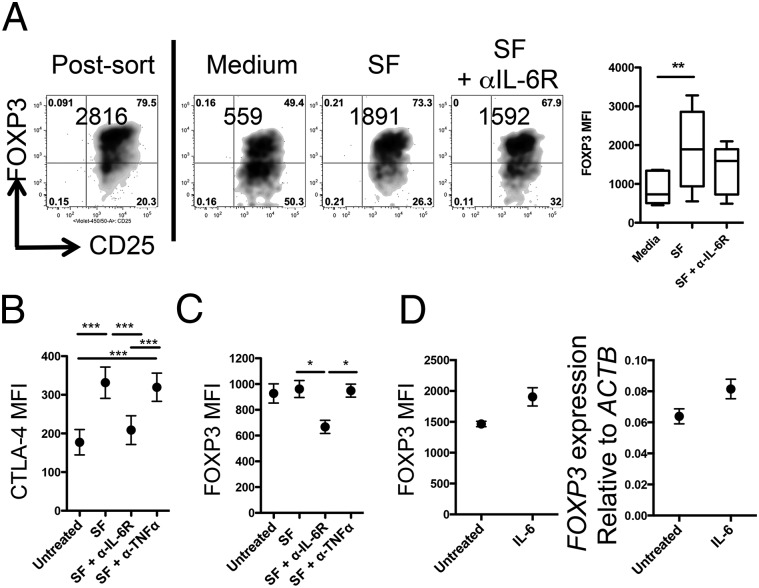
IL-6R signaling by SF can positively regulate FOXP3 and CTLA-4 expression. (**A**) Tregs were isolated from the SF of a JIA patient and cultured for 0 (*left flow cytometry plot*) or 18 h (*right plots*) in the presence of medium alone, 10% SF, or 10% SF in the presence of 10 μg/ml anti–IL-6R (TOC). Cells were stained for CD25 and FOXP3 expression. Numbers represent the median fluorescence intensity (MFI) of FOXP3 expression. Summary box and whiskers plot displays the FOXP3 MFI under the various culture conditions at the 18-h time point. Bars represent medians plus quartiles. Statistical analysis by Friedman test, with Dunn multiple comparisons, *n* = 5. (**B**–**D**) Tregs from control blood were isolated and cultured for 48 h either in media alone, 10% SF, 10% SF + 10 μg/ml anti–IL-6R (TOC), or 10% SF + 10 μg/ml anti–TNF-α before analysis of total CTLA-4 (B) or FOXP3 (C) protein expression. (D) Tregs were cultured with or without 10 ng/ml IL-6 for 48 h before analysis of FOXP3 expression by flow cytometry (*left panel*) or quantitative PCR (*right panel*). Bars represent mean ± SEM. Statistical analysis by repeated-measures ANOVA, with Tukey’s multiple comparisons test (*n* = 3, *p < 0.05, **p ≤ 0.01, ***p = 0.001). All data are representative of at least three independent experiments.

IL-6 can redirect the differentiation of Tregs into the Th17 lineage in mice (24, 25) and is thought to do this through a STAT3-dependent mechanism. Given that SF was a potent STAT3 activator, it was unexpected that SF could stabilize FOXP3 expression in synovial Tregs. We hypothesized that neutralization of IL-6 signaling in SF may act to further stabilize FOXP3 expression by removing STAT3 signals within the joint. Surprisingly, cotreatment of SF with TOC resulted in a trend for a reduction in FOXP3 expression, suggesting that IL-6 signaling does not negatively regulate committed Treg FOXP3 expression. In fact, IL-6R signaling acted as a positive regulator of CTLA-4 expression on healthy Tregs (Fig. 6B), and blockade of IL-6R signaling within SF resulted in a small but statistically significant reduction in healthy Treg FOXP3 expression (Fig. 6B, 6C). Interestingly neutralization of TNF-α had no effect on either protein. Culture of control Tregs with or without rIL-6 demonstrated that the small increase in FOXP3 protein was likely transcriptionally driven, because mRNA levels of *FOXP3* were also slightly elevated (Fig. 6D). These findings suggest that IL-6 can exert proregulatory properties at the inflamed site, as has been reported in the generation of regulatory B cells in mice (26), but that other unidentified factors exist in SF that additionally promote Treg FOXP3 protein stability.

## Discussion

In this study, we have used-high throughput TCR sequencing to reveal the relationships between CD4^+^ T cell subsets in JIA patients and have demonstrated that the stability of human synovial Treg FOXP3 expression is highly sensitive to the local environment. Although our data establish that FOXP3 protein expression is susceptible to downregulation, intriguingly this did not appear to be paralleled by a *FOXP3* transcriptional downregulation. Such uncoupling of FOXP3 protein expression from transcriptional activities has been reported previously (27), and was explained by the fact that FOXP3 can be targeted for degradation by the ubiquitin/proteasome pathway (27, 28). Culture of SF Tregs with cycloheximide did not appear to accelerate FOXP3 loss, suggesting that FOXP3 protein is rapidly turned over. Although this would be difficult to show using limited primary cells from the inflamed site in humans, the kinetics of the loss of FOXP3 protein in this report are consistent with these previously published findings.

Our results suggest, unlike in those previous studies, that exposure to inflammatory signals (in our case, contained within SF) may act to stabilize FOXP3 expression. These data strongly suggest that signals within the inflamed joint act to promote FOXP3 protein stability. The identity of the factor(s) and pathways responsible are as yet unidentified, but we were able to rule out IL-6 and TNF-α as significantly contributing to FOXP3 downregulation. Our finding that IL-6 did not induce FOXP3 downregulation, in contrast with a recent study (27), may also be explained by the source and preconditioning of Tregs used. Indeed, the studies by van Loosdregt et al. (27) and Chen et al. (28) used a Treg activation step with anti-CD3 and anti-CD28 in the presence of IL-2 before exposure to inflammatory cytokines. In addition, many of the studies were performed on murine isolated Tregs or with in vitro differentiated human Tregs, the TSDR methylation status and FOXP3 stability of which are likely to be different from ex vivo synovial Tregs. It is also known that IL-6 can activate STAT5 (29), which may explain how it can weakly positively modulate FOXP3 expression, as observed in this study. Therefore, the differentiation and/or activation state of Tregs may be important for dictating the sensitivity to inflammatory signals. Our demonstration, however, that Tregs phosphorylate STAT3 in response to both SF and IL-6 suggest no issue with IL-6 sensitivity. Furthermore, our data suggest that the therapeutic mechanism of TOC is unlikely to work through boosting Tregs, because IL-6 was shown to exert a positive effect on CTLA-4 expression.

IL-2 is known to be a positive transcriptional regulator of FOXP3 expression (30), and the observation that IL-2 and other common γ-chain cytokines failed to restore FOXP3 expression adds weight to our conclusion that degradation, not transcriptional downregulation, was the major mechanism behind FOXP3 loss. This finding should be of interest to those wishing to use Tregs as therapy for autoimmune disorders. Although most studies designed to generate and expand Tregs in vitro for use in clinic have used epigenetic motifs, such as the TSDR, to validate Treg stability, this does not consider the possibility that posttranslational mechanisms may negatively impact on Tregs at inflamed sites. It is known that cyclin-dependent kinase 2 can regulate the stability of FOXP3 protein (31), raising the possibility that factors affecting cell cycle/division may also affect FOXP3 stability. As such, delineating the signals responsible for stabilizing FOXP3 could open up new avenues for therapeutic intervention. In particular, degradation of FOXP3 may be the desired goal in tumor biology, to allow the propagation of antitumor responses, as has been demonstrated with anti-CTLA4 therapies (32). In contrast, stabilizing FOXP3 may be important for resolving chronic inflammation, as has been demonstrated in a colitis model (27). Therefore, a detailed investigation of the signaling pathways engaged by exposure to, and/or withdrawal from, SF would help to determine the mechanisms at play.

Although the Treg community has taken as a rule of thumb that stable FOXP3 expression is essential to Treg lineage stability, our data investigating the TCR repertoires in human arthritis suggest a more nuanced situation. It is strikingly apparent that there is limited overlap between the Tcon and Treg repertoires, whether it is in blood or at the inflamed site. This was also largely true for the repertoires of atypical Treg populations CD25^−^FOXP3^+^ and CD25^+^FOXP3^−^ with Tcons. This suggests that FOXP3 or CD25 downregulation does not necessarily lead to reprogramming of Tregs; moreover, it suggests a dynamic picture where expanding Treg clones likely compete for limited resources, which may lead to fluctuations in FOXP3 and CD25 expression. In this study, we must also consider the possibility that CD25^+^FOXP3^−^ T cells could also upregulate FOXP3^+^, that is, that FOXP3 levels can both rise and fall within a given antigenic niche, possibly as a result of competition for limited resources. This interpretation would be in keeping with a recently proposed feedback control perspective model for Tregs/FOXP3, which places more emphasis on the function of FOXP3 as a negative feedback component of T cell activation, rather than on its role in determining a distinct T cell lineage (33).

Although FOXP3 loss has always been considered to be indicative of Treg destabilization, the dynamics of FOXP3 expression in individual cells is poorly understood. Evidence for a stable Treg lineage has been published in mice, where the use of genetic reporter systems have shown that the majority of the Treg subset is stable (34), and that any apparent conversion is a result of promiscuous FOXP3 expression by conventional T cells, rather than reprogramming of Tregs (35). The data presented in this article can now start to address this situation in humans. Given that the synovial Treg repertoire most closely aligns with blood Tregs, rather than any other population, and that Treg and Tcon repertoires are near exclusive in blood and SF, our data give some weight to a (largely) stable Treg lineage model. Similar recent findings between Tregs and Tcons have also been reported in human inflammatory bowel diseases, where minimal Treg and Tcon repertoire overlap was observed (21), suggesting that the Treg/Tcon repertoire divergence extends beyond blood and SF in humans. This lineage divergence in itself does not preclude the existence of hybrid Treg/Tcon phenotypes, the likes of which have been observed in mouse (36), as well as within memory (37) and CD161-expressing Tregs in humans (38, 39), because such cells have been shown to retain their Treg functions.

Further exploratory research into the repertoire of Tregs in the thymus compared with other tissues would help to ascertain the validity of the Treg lineage model in humans. It remains controversial how Tregs are selected in the thymus, with some evidence to suggest that TCRs with high affinity for self-Ag are positively selected into the Treg lineage (40); however, a recent study has challenged this view and suggested that the same self-peptide can select Tcons or Tregs with identical TCRs, but that the affinity of the interaction determined whether the Treg and Tcon repertoires were similar or different, implicating negative selection as an important player (41). Clearly such in vivo studies in humans are not feasible, but it would be highly informative to interrogate the thymic repertoires in different T cell subsets, in particular, Tregs and Tcons, to validate whether the repertoires are also divergent early in their differentiation.

In summary, we have demonstrated that in human autoimmune arthritis the TCR repertoires of Tregs and Tcons show high exclusivity, both in blood and at the inflamed site, suggesting that regulatory-like T cells occupy discrete antigenic niches. The expansion of Treg clones, however, likely leads to interclonal and intraclonal competition for positive regulators of CD25 and FOXP3 expression within SF, resulting in some Tregs downregulating these proteins. Given FOXP3’s central role in Treg biology, the future identification of the signaling molecule(s) in SF that stabilizes FOXP3 protein is likely to be of high therapeutic interest.

## Supplementary Material

Data Supplement
