# Supplementary material for: Synovial Regulatory T Cells Occupy a Discrete TCR Niche in Human Arthritis and Require Local Signals To Stabilize FOXP3 Protein Expression
Source: J Immunol. 2015 Nov 11;195(12):5616–24. doi: 10.4049/jimmunol.1500391 (PMC4671090; doi:10.4049/jimmunol.1500391)
Supplement: Data Supplement [file JI_1500391.zip › JI_1500391_Supplemental_Material_1.pdf]

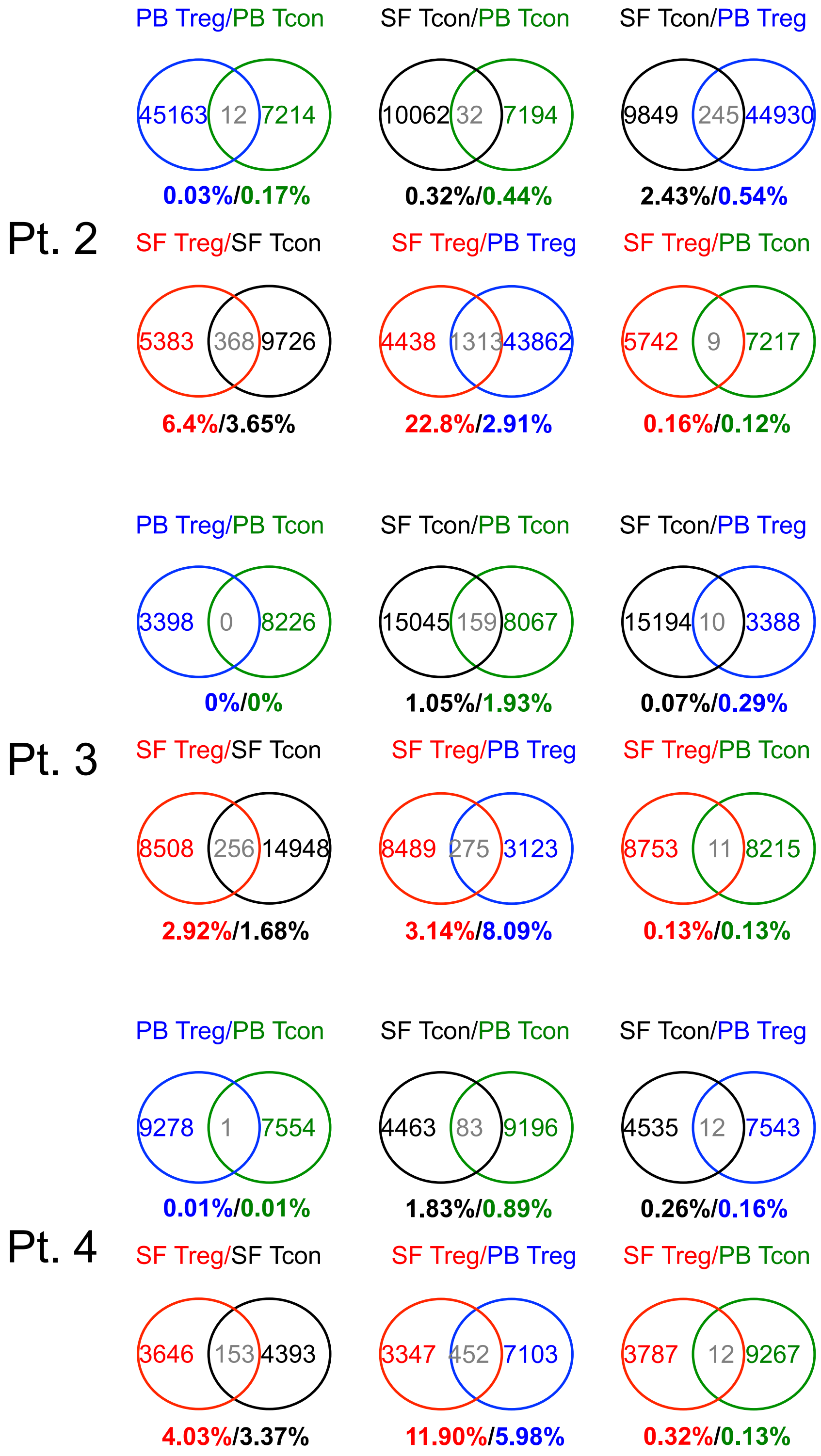

Supplementary Figure 1 (related to Figure 1)

Venn diagrams depict the sharing of unique clones (at nucleotide level) between each sorted T-cell subset from Patients 2, 3 and 4. The percentage figure represents the % of sequences shared as a total of that T-cell compartments repertoire. Blue = PB Treg, Green = PB Tcon, Black = SF Tcon, Red = SF Treg. Shared unique sequences are depicted in grey.

A

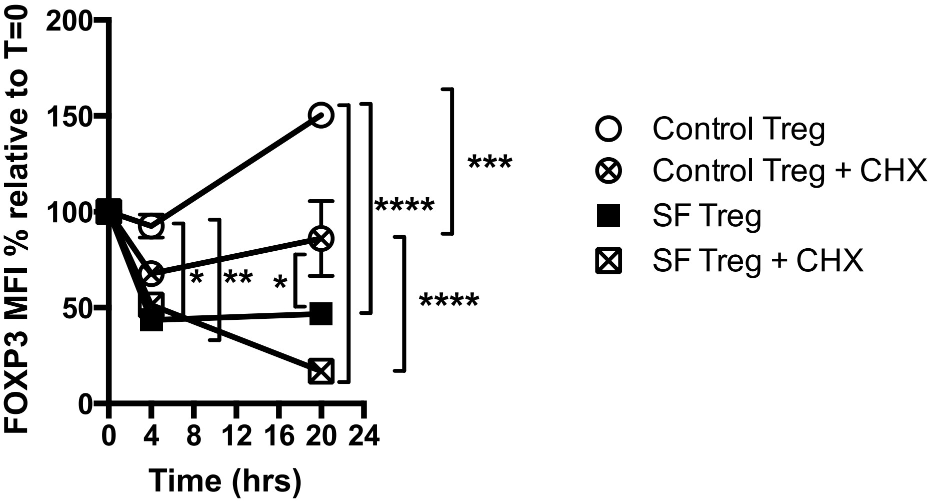

B

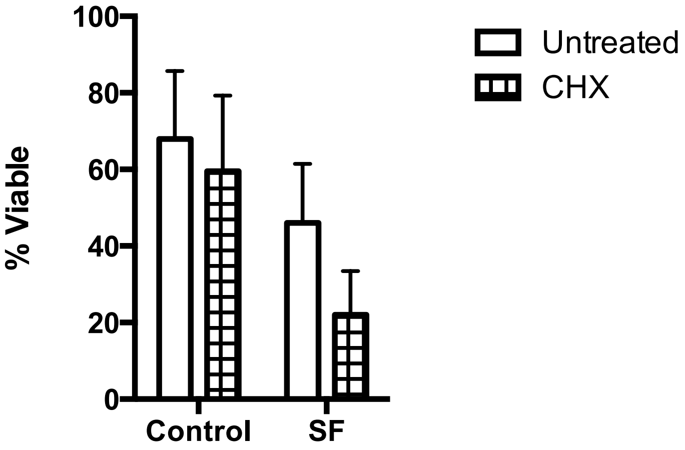

Supplementary Figure 2

Control Treg or synovial fluid (SF) Treg of JIA patients were sorted ($CD4^{+}CD25^{hi}CD127^{lo}$) and cultured for 0, 4 or 20hrs in the presence of absence of 50μg/ml cycloheximide (CHX). Cells were analysed for FOXP3 protein expression by flow cytometry, n=3 (A). Statistical testing by Two-way Anova. * = p<0.05, ** = p<0.01, **** = p<0.001 and **** = p<0.0001. (B) Cellular viability at the 20hr time point was assessed by staining with a fixable viability dye and analysis by flow cytometry.

Supplemental Table 1

Patient	Diagnosis	MTX	Steroids	Active Joints	ESR	Figures
**1**	Pers-Oligo	Yes	Yes	2	30	1A-D
**2**	Pers-Oligo	No	Yes	1	19	1B, 1D, S1
**3**	Poly-JIA	Yes	No	7	76	1B, 1D, 3B, S1
**4 (1)**	Ext-Oligo	Yes	No	2	N/A	1B, 1D, 3B-C, S1
**4 (2)**	Ext-Oligo	Yes	No	3	N/A	3A-B, S2
**5**	Poly-JIA	N/A	N/A	N/A	N/A	2A-E, T1, ST1
**6**	Ext-Oligo	No	No	7	30	2B-E
**7**	Ext-oligo	No	No	2	N/A	3B-C, S2
**8**	Poly-JIA	No	No	9	25	3B-C
**9**	Poly-JIA	Yes	No	N/A	N/A	3B
**10**	Poly-JIA	No	Yes	N/A	N/A	3B-D, 4A, 4C-D, 4F
**11**	Ext-Oligo	Yes	No	4	46	3B-C
**12**	Ext-Oligo	No	Yes	4	N/A	3B, S2
**13**	Pers-Oligo	No	Yes	1	N/A	3A-B
**14**	Pers-Oligo	No	Yes	4	9	3B
**15**	Pers-Oligo	No	No	1	N/A	4D, 4F
**16**	Poly-JIA	No	No	N/A	6	4D-F
**17**	Poly-JIA	Yes	No	2	13	4D-F
**18**	Poly-JIA	No	No	2	N/A	4D-F
**19**	Poly-JIA	N/A	N/A	N/A	N/A	4D-F
**20**	Pers-Oligo	Yes	Yes	1	N/A	4B
**21**	Ext-Oligo	Yes	Yes	N/A	N/A	6A
**22**	Pers-Oligo	No	Yes	1	14	6A
**23**	Ext-Oligo	Yes	No	4	6	6A
**24**	Pers-Oligo	No	Yes	1	N/A	6A
**25**	Poly-JIA	No	No	4	106	6A

MTX = Methotrexate
ESR = erythrocyte sedimentation rate mm/hr
Pers-Oligo = persistent oligoarticular JIA
Ext-Oligo = extended oligoarticular JIA
Poly-JIA = Polyarticular JIA
N/A = data not available

Supplemental Table 2

Clone	Nucleotide	Total	P1 Treg	P2 Treg	P3 Treg	Tcon
1	TCGGCTGCTCCCTCCCAAACATCTGTGTACTTCTGTGCCAGCAGTTCTGACTTCGCTAGCGGGACCTACGAGCAGTACTTCGGGCCG	35916	0.658	1.164	2.541	0
2	AATTCCCTGGAGCTTGGTGACTCTGCTGTGTATTTCTGTGCCAGCAGCCAAGGGGGGACTTCTGGAAACACCATATATTTTGGAGAG	30623	1.491	1.069	1.034	0
3	GGCTGGAGTTGGCTGCTCCCTCCCAGACATCTGTGTACTTCTGTGCCAGCAGTTACTCGTTCTAGGCAGCCCCAGCATTTTGGTGAT	28128	1.312	0.869	1.144	0
4	CCGCTCAGGCTGGAGTTGGCTGCTCCCTCCCAGACATCTGTGTACTTCTGTGCCAGCAGTTCAGGTCAGCCCCAGCATTTTGGTGAT	22628	1.893	0.169	0.584	0.004
5	CTGGAGCTTGGTGACTCTGCTGTGTATTTCTGTGCCAGCAGCCAGGAACAGTGGGCCAGATTCTCCTACGAGCAGTACTTCGGGCCG	22550	0.017	1.709	0.919	0
6	CTCACTCTGGAGTCCGCTACCAGCTCCCAGACATCTGTGTACTTCTGTGCCATCCGAGGGACTAGCTACGAGCAGTACTTCGGGCCG	21718	0.013	1.733	0.788	0.001
7	TGAACGCCTTGGAGCTGGACGACTCGGCCCTGTATCTCTGTGCCAGCAGCTACGGACCTTCTCGGGAACACCATATATTTTGGAGAG	20068	0.435	0.623	1.376	0
8	CACACCCTGCAGCCAGAAGACTCGGCCCTGTATCTCTGCGCCAGCAGCCAAGATCTGGGGTGGGATGAAAAACTGTTTTTTGGCAGT	19490	0.644	0.463	1.249	0
9	ATCCTGGAGTCGCCCAGCCCCAACCAGACCTCTCTGTACTTCTGTGCCAGCAGTTTCCAGACAGGGAGTGGCTACACCTTCGGTTCG	17557	0.847	0.599	0.618	0
10	TGTCGGCTGCTCCCTCCCAGACATCTGTGTACTTCTGTGCCAGCAGGGACCCCGGACGGCAACCGCTACGAGCAGTACTTCGGGCCG	17493	0.019	1.310	0.725	0
11	CTGTCGGCTGCTCCCTCCCAGACATCTGTGTACTTCTGTGCCAGCAGCCACCGAGGATCGGCGAACACTGAAGCTTTCTTTGGACAA	17379	0.049	1.334	0.649	0
12	CAGCCCTCAGAACCCAGGGACTCAGCTGTGTACTTCTGTGCCAGCAGTCCAGCGGGAGGATTTGGTTACGAGCAGTACTTCGGGCCG	17143	0.223	1.018	0.784	0.001
13	CTGCTGTCGGCTGCTCCCTCCCAGACATCTGTGTACTTCTGTGCCAGCAGCCGCGTTCCTACAACCTACGAGCAGTACTTCGGGCCG	16574	0.331	0.506	1.177	0
14	ATCCGGTCCACAAAGCTGGAGGACTCAGCCATGTACTTCTGTGCCAGCAGTCGTCCCCCAAATACCTACGAGCAGTACTTCGGGCCG	16358	0.361	0.550	1.065	0
15	CTGGAGTTGGCTGCTCCCTCCCAGACATCTGTGTACTTCTGTGCCAGCAGACCGATAGACAGGGAGGGTGGCTACACCTTCGGTTCG	15354	0.232	0.651	0.965	0
16	CACATCAATTCCCTGGAGCTTGGTGACTCTGCTGTGTATTTCTGTGCCAGCAGCTGGACAGGTGACACTGAAGCTTTCTTTGGACAA	12801	0.061	0.780	0.682	0
17	CTTGGAGCTGGACGACTCGGCCCTGTATCTCTGTGCCAGCAGCTTAAACCCGGGACAGGGGTCCCTCAGCCCCAGCATTTTGGTGAT	12675	1.032	0.106	0.344	0.005
18	GTGAACGCCTTGGAGCTGGACGACTCGGCCCTGTATCTCTGTGCCAGCAGCTTGAAGGGGGCAGGGGAGACCCAGTACTTCGGGCCA	12460	0.151	0.531	0.823	0
19	GTGAACGCCTTGGAGCTGGACGACTCGGCCCTGTATCTCTGTGCCAGCAGCTTGCAGGGGGCCGCTGAAAAACTGTTTTTTGGCAGT	12084	1.042	0.136	0.226	0
20	ACGATCCAGCGCACAGAGCAGCGGGACTCGGCCATGTATCGCTGTGCCAGCAGCTCACGAGATAACACTGAAGCTTTCTTTGGACAA	11101	0.016	0.817	0.472	0
21	AAGATCCGGTCCACAAAGCTGGAGGACTCAGCCATGTACTTCTGTGCCAGCAGTGGACAGGGAGGCTATGGCTACACCTTCGGTTCG	5997	0.467	0.127	0.098	0.002
22	ATCCAGCGCACACAGCAGGAGGACTCGGCCGTGTATCTCTGTGCCAGCAGCCGAGGGAACGGGAGGTTCGAGCAGTACTTCGGGCCG	4402	0.463	0.003	0.042	0.001
23	CCGCTCAGGCTGGAGTTGGCTGCTCCCTCCCAGACATCTGTGTACTTCTGTGCCAGCCAATCGAATGAAAAACTGTTTTTTGGCAGT	3867	0.010	0.034	0.109	0.476
24	GTCCACGGAGTCAGGGGACACAGCACTGTATTTCTGTGCCAGCTCTCAAAGAGAGGCAGCCATAATGAAAAACTGTTTTTTGGCAGT	3604	0	0.002	0.099	0.509
25	AGATCCAGCGCACAGAGCAGGGGGACTCAGCCGCGTATCTCCGTGCCAGCAGCTTATGCAACTAATGAAAAACTGTTTTTTGGCAGT	2775	0.005	0	0.130	0.311
26	CTGGAGTCGCCCAGCCCCAACCAGACCTCTCTGTACTTCTGTGCCAGCAGGCTAGGGGGGGTCCGGACTGAAGCTTTCTTTGGACAA	2203	0	0	0.026	0.363
27	GCTGGGGTTGGAGTCGGCTGCTCCCTCCCAAACATCTGTGTACTTCTGTGCCAGCAGTAACTCTTTGAAAAACTGTTTTTTGGCAGT	2078	0.060	0	0.009	0.268
28	ATCCGGTCCACAAAGCTGGAGGACTCAGCCATGTACTTCTGTGCCAGCAGCCTCAAAAACAGGGGCACTGAAGCTTTCTTTGGACAA	1980	0	0	0.007	0.349
29	CTGGAGTTGGCTGCTCCCTCCCAGACATCTGTGTACTTCTGTGCCAGCAGATCCGGGACAGGGGCCTACGAGCAGTACTTCGGGCCG	1922	0	0	0.027	0.310
30	GAGTCGCCCAGCCCCAACCAGACCTCTCTGTACTTCTGTGCCAGCAGTTTGGGACAGCCCTCCTACAATGAGCAGTTCTTCGGGCCA	1866	0	0	0.064	0.248
